# Symptoms and pathophysiology of post-acute sequelae following COVID-19 (PASC): a cohort study

**DOI:** 10.1016/j.ebiom.2025.105792

**Published:** 2025-05-30

**Authors:** Olivier Robineau, Sophie Hüe, Mathieu Surenaud, Cédric Lemogne, Céline Dorival, Emmanuel Wiernik, Sebastien Brami, Jerome Nicol, Xavier de Lamballerie, Hélène Blanché, Jean-François Deleuze, Céline Ribet, Marcel Goldberg, Gianluca Severi, Mathilde Touvier, Marie Zins, Yves Levy, Jean-Daniel Lelievre, Fabrice Carrat

**Affiliations:** a Sorbonne Université, Inserm, Institut Pierre-Louis d'Epidémiologie et de Santé Publique, Paris, France; b EA2694, Centre Hospitalier de Tourcoing, Univ Lille, France; c Faculté de Médecine, INSERM U955, Team 16, Vaccine Research Institute, Université Paris-Est Créteil, Créteil, France; d Center for Research in Epidemiology and StatisticS (CRESS), Université Paris Cité and Université Sorbonne Paris Nord, Inserm, INRAE, Paris, France; e Service de Psychiatrie de l’adulte, AP-HP, Hôpital Hôtel-Dieu, Paris, France; f Population-based Epidemiological Cohorts, UMS 11, Université de Paris Cité, Université de Paris Saclay, Université de Versailles St Quentin, Inserm, Villejuif, France; g Unité des Virus Emergents, UVE: Aix Marseille University, IRD 190, Inserm 1207, IHU Méditerranée Infection, Marseille, France; h Fondation Jean Dausset-CEPH (Centre d’Etude du Polymorphisme Humain), Paris, France; i CESP UMR1018, Paris-Saclay University, UVSQ, Inserm, Gustave Roussy, Villejuif, France; j Department of Statistics, Computer Science, Applications “G. Parenti”, University of Florence, Italy; k Sorbonne Paris Nord University, Inserm U1153, Inrae U1125, Cnam, Nutritional Epidemiology Research Team (EREN), Epidemiology and Statistics Research Center – University of Paris (CRESS), Bobigny, France; l Assistance Publique-Hôpitaux de Paris, Groupe Henri-Mondor Albert-Chenevier, Service de maladies Infectieuses et Immunologie Clinique, Créteil, France; m Département de Santé Publique, Hôpital Saint-Antoine, APHP, Paris, France

**Keywords:** SARS-CoV-2, PASC, Long COVID, Biomarkers, Post-infectious symptoms, Postacute symptoms following COVID-19

## Abstract

**Background:**

Several studies reported long-term consequences of severe COVID-19. However, pathophysiological mechanisms of Post-Acute Sequelae following COVID-19 (PASC) in patients with mild acute COVID-19 have been less investigated. Specifically, the link between PASC and immuno-inflammatory abnormalities is inconsistent in the literature. The hypothesis that different pathophysiological mechanisms could explain the persistent symptoms needs to be explored.

**Methods:**

The COPER cohort is a prospective study that included participants with PASC and with a history of COVID-19 without persistent symptoms. None were hospitalised for COVID-19. Participants underwent two home visits six months apart for biological sample collection and completed questionnaires on medical history, infection, vaccination, symptoms, and mental health. The study analysed association between persistent symptoms and 14 blood biomarkers, comparing participants with PASC with recovered participants.

**Findings:**

Between June and November 2022, 1000 participants were included in the study, 199 were excluded due to missing data or sample (35), SARS-CoV-2 infection less than 3 months (36) or lack of known SARS-CoV-2 infection and negative serology (128), with two groups analysed: recovered (n = 490), PASC (n = 311). Participants with PASC were more frequently women, had a higher BMI and a median number of 3 persistent symptoms, with common symptoms being asthenia, dyspnoea, cough, and sleep disorders. Biological analysis revealed significant associations between certain PACS symptoms and biomarkers of viral activation (IFNγ, IP-10), COVID-19 severity (CD163) and vascular activation (VCAM-1, ICAM-1), mainly in subjects whose symptoms had lasted less than a year. However, these associations did not persist over time.

**Interpretation:**

The results suggest a polymorphic and dynamic pathophysiology according to symptoms and time since infection. Other hypotheses, beyond those related to persistent inflammation, should be explored.

**Funding:**

French Ministry of Health and Prevention and the French Ministry of Higher Education, Research and Innovation.

**Research in context**

**Evidence before this study**

In December 2022, we conducted a PubMed search to identify articles on the pathophysiology of post-acute symptoms following COVID-19 (PASC) and critically review them with the goal of highlighting the proposed hypotheses and potential biomarkers associated with these hypotheses. The main pathophysiological hypotheses studied included viral persistence (persistent antigens and RNA in blood or tissues), immune dysregulation and inflammation (B- and T-cell activation, T-cell exhaustion, and inflammatory pathway biomarkers like IL-6, IL-1β, TNF-α), endothelial dysfunction, coagulopathies, autoimmunity, and mitochondrial dysfunction.

While these studies provided evidence supporting these hypotheses, significant biases affected their findings. Most excluded outpatient participants, who represent the majority of PASC cases. Studies that included outpatients often had small sample sizes, and/or did not differentiate them from non-hospitalised patients, and/or lacked control groups. Inconsistent results between studies were common, with some showing associations between biomarkers and PASC while others did not. Statistical analyses often failed to adjust for confounding factors such as age, sex, weight, or time since infection. Additionally, most studies treated patients with PASC as a single homogeneous group. A rare exception was a comparative study focusing on individuals with neurological complaints. These limitations highlight the need for more robust research to better understand links between the biomarker, the pathophysiology of PASC and the polymorphism of this condition.

**Added value of this study**

The study, initiated in 2022 but conceptualised in mid-2020, hypothesises that the variability in clinical symptoms can be attributed to multiple underlying mechanisms. To investigate this, we assembled a sufficiently large cohort to examine symptoms, the biomarkers associated with them, and the progression of both symptoms and biomarkers over time.

Our models took into account variables that could influence biomarker values, including age, sex, BMI, and date of infection. The findings reveal that certain biomarkers are associated with specific symptoms, while others show no such connection, with these associations influenced by the time elapsed since acute infection. Additionally, the results indicate that while the resolution of symptoms is occasionally linked to changes in biomarkers, this is not the case in most instances. Notably, the strength of associations between biomarkers and symptoms tends to diminish over time.

**Implications of all the available evidence**

The literature review was updated in February 2025. Studies identifying biomarkers to define PASC remain insufficient to establish clear pathophysiological pathways explaining symptomatology. Biomarkers from the acute phase in patients with severe illness have been shown to predict the risk of persistent symptoms. However, markers of chronic inflammation, viral or antigenic persistence, and tissue damage have been reported with varying consistency. These findings, along with our own work, confirm the complexity of PASC. With the exception of recent studies on neurological symptoms and severe pulmonary complaints, few investigations have thoroughly explored PASC biomarkers in relation to specific symptoms and time. Our findings highlights that biomarker variations depend on both the symptoms presented and the time since infection, emphasising the need to take these factors into account when interpreting biomarker data for diagnostic purposes. The number of identified biomarkers remain low, and the need for further longitudinal omics-integrated studies remains crucial. From a therapeutic perspective, if these biomarkers have a causal relationship with specific symptoms, the effectiveness of treatments targeting these pathways may vary over time and between patients, necessitating a personalised approach to management.

## Introduction

Post-acute sequelae of severe acute respiratory syndrome coronavirus 2 (SARS-CoV-2; PASC), commonly referred to as long COVID, has become a significant public health concern. The prevalence of this condition ranges from 3% to over 30%, depending on the methodologies and populations studied.^1^, ^2^, ^3^, ^4^ Even years after infection, some patients continue to experience symptoms and a reduced quality of life, confirming that this post-infectious condition remains a significant public health challenge. This also underscores the need for further research to better understand its mechanisms and to prepare for similar symptom profiles that may arise in future epidemics. The pathophysiological origins of PASC remain elusive, and the relationship between biological abnormalities and symptoms is difficult to establish mechanistically. The main pathophysiological pathways explored in PASC include chronic inflammation and immune dysregulation, with persistent activation of inflammatory pathways potentially contributing to prolonged symptoms. Another key hypothesis involves viral or antigenic persistence, whereby remnants of SARS-CoV-2 or its proteins continue to stimulate the immune system, resulting in ongoing symptoms. Additionally, endothelial dysfunction and microvascular damage have been implicated, as vascular abnormalities may underpin the neurological, cardiovascular, and respiratory symptoms observed in affected individuals.^5^ Individuals hospitalised for COVID-19 appear to be at the highest risk of developing PASC; however, a substantial number of outpatients with a history of “mild COVID-19” also experience this condition. Risk factors for PASC include female sex, underlying chronic diseases, older age, overweight, tobacco use, psychological factors, and a high symptom burden during the acute phase.^6^, ^7^, ^8^

Moreover, PASC exhibits considerable heterogeneity in terms of symptoms and impacts. Recent studies in the general population have begun to distinguish clinical patterns of PASC, but there are currently no published data linking persistent symptoms to specific pathophysiological mechanisms.^9^ Consequently, there is a pressing need to understand the heterogeneity of PASC and decipher whether distinct pathophysiological origins underlie specific complaints.^10^

In this study, we present findings from a cohort of individuals with PASC and participants who recovered from COVID-19, drown from general population cohorts in France. The aim was to characterise PASC in terms of symptoms and to explore potential associations with a range of blood biomarkers, stratified by time since infection.

## Methods

### The COPER cohort

**Fig. 1:**
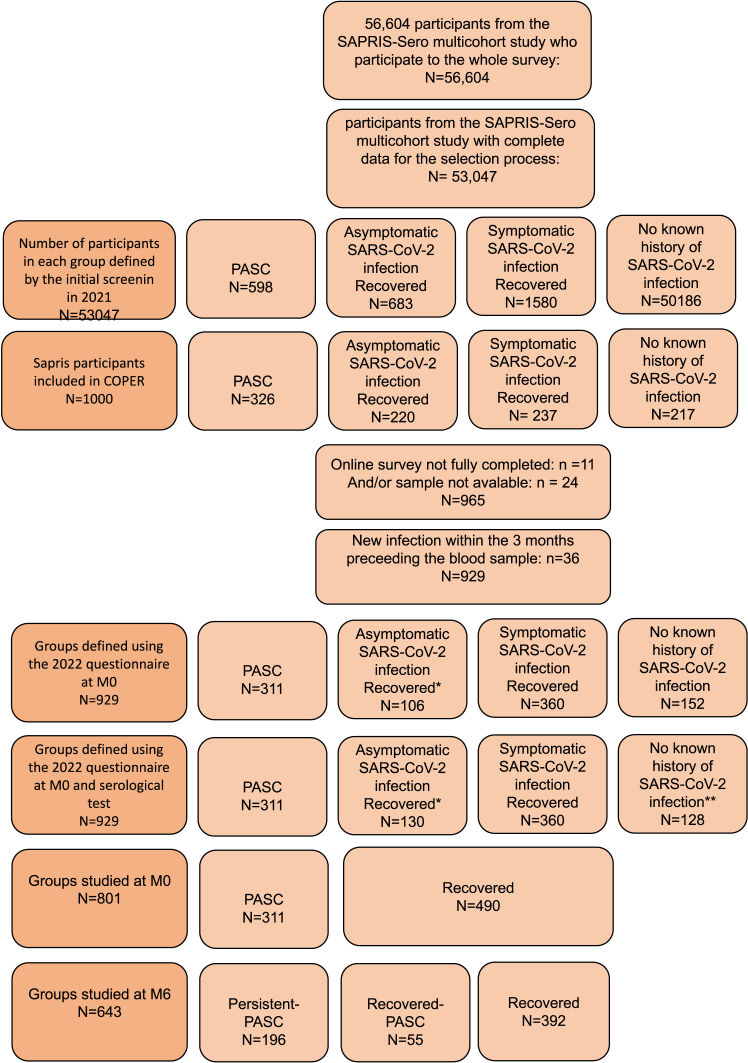
**Flow chart of the COPER survey.** ∗Defined by the absence of symptoms but a history of positive serology and/or a positive PCR test; ∗∗defined by the absence of SARS-CoV-2 infection by RT-PCR and the absence of positive serological test made on serum taken at the inclusion visit (M0).

The COPER cohort is a prospective study nested within the SAPRIS-Sero cohort, encompassing three general adult population-based French cohorts (E3N-Generations, Constances, and Nutrinet-Santé).^11^ The aim of the SAPRIS-Sero cohort was to quantify the cumulative incidence of SARS-CoV-2 infection in the French population and to estimate the prevalence, natural evolution, and risk factors for symptoms following COVID-19. Data on symptoms suggestive of SARS-CoV-2 infection along with serological and PCR results were collected between February 2020 and October 2021 from 53,047 participants with complete data (i.e., serological results and questionnaires fulfilled). The questionnaires included a self-assessment covering the symptoms experienced in 2020 and 2021, their duration or how long they had lasted, whether the symptoms were ongoing, and whether they had been present prior to February 2020. Symptoms were selected based on those known to occur during the acute phase of infection, as well as those most frequently reported by patients during medical consultations in the months leading up to the study (May–June 2020). A sampling screening procedure was then applied to identify eligible participants for the COPER cohort based on these biological results and the presence of persistent symptoms at the last follow-up questionnaire. We included participants with asymptomatic SARS-COV-2, participants with COVID-19 with and without persistent symptoms, and individuals without known history of SARS-CoV-2 infection (see Supplementary appendix p 2 for the detailed selection process). As a result of the screening process, eligible participants that were *a priori* identified according to the presence or the absence of persistent symptoms and their history of SARS-CoV-2 infection, were invited to take part to the COPER cohort via an online survey. One thousand of them provided written informed consent (see flowchart Fig. 1). They subsequently underwent two home visits, 6 months apart (M0 and M6 visits), during which blood, saliva, and urine samples were collected. At each visit, participants completed self-administered questionnaires detailing symptoms experienced at the time of the visit and over the preceding 15 days. Based on these responses, participants were reclassified to account for recent SARS-CoV-2 infection or the resolution of symptoms since screening. Additional questionnaires covered medical history, SARS-CoV-2 infection and vaccination status, and cognitive function. Anxiety and depression were assessed using the CES-D (score >19) and GAD-7 (score > 10) scales. Hyperventilation syndrome was considered if the Nijmegen score exceeded 22.

### Groups definitions

This analysis focused on participants of the COPER cohort most recent documented SARS-CoV-2 infection had occurred more than three months prior to the M0 visit. The *Recovered* group was defined by a known positive serological or PCR test, with no persistent symptoms reported the M0 or during the preceding 15 days. Individuals reporting at least one persistent symptom that emerged following the acute SARS-CoV-2 infection and remained present in the 15 days prior to M0 were classified as the *PASC* group. Questionnaires also assessed whether participants had sought medical advice for these symptoms and whether a physician had associated the symptoms with SARS-CoV-2 infection. These data enabled a sensitivity analysis in a subgroup referred to as *medically-validated-PASC*.

To assess the evolution of biomarkers in relation to symptoms progression at M6, we further categorised individuals within the PASC groups. Those with at least one persistent symptom at M6 formed the *Persistent PASC* group, while those with no persistent symptoms at M6 were classified as *Recovered PASC*.

### Blood biomarker analysis

We analysed 14 different blood biomarkers, including cytokines, chemokines, immune checkpoints, cell adhesion molecules, and markers of macrophage activation, intestine cellular injury, and vascular damages. These biomarkers were chosen in an exploratory manner based on the literature and the main research hypotheses regarding the origin of persistent symptoms. They are included in the major pathophysiological mechanisms associated with the activation of the immune system by SARS-CoV-2, as described in patients with severe forms of COVID-19. They also describe tissue injurie and endothelial dysfunction (Supplementary Table S1, Appendix 1 p5). Biomarkers were quantified via the ELLA rapid detection enzyme-linked immunosorbent assay (ELISA) microfluidics platform (Bio-Techne, UK). The ELLA platform is a rapid cytokine detection system based on four or six parallel singleplex microfluidics ELISAs run in triplicate or duplicate within cartridges following the manufacturer’s instructions. The concentrations of 14 blood biomarkers were determined via three different cartridges. One multiplex cartridge for IL-1β, IFNγ, IL-6, IL-8, TNFα and TRAIL (SPCKC-PS-006898, Bio-Techne); one multianalyte cartridge for PD-L1, IP-10, IL-18 and FABP (SPCKC-PS-006872, Bio-Techne); and another multianalyte cartridge for ICAM-1, VCAM-1, CD163 and PDGF-BB (SPCKE-PS-006899, Bio-Techne) were used. Extrapolated calculated concentrations below the lower limit of quantification (LLOQ) were considered only if the sample coefficient of variation (CV) was lower than 20%.

### Determination of symptom associations among participants with PASC

To examine associations between symptoms, we constructed a correlation matrix and represented symptom interrelations using a network structure, in which each symptom (node) is connected to others via edges. The edges represent correlation estimates among the nodes. We then used an L1-regularised partial correlation method to estimate the network and the significance of the links.^12^ Compared with a simple correlation network, this method is known to better fit the causal architecture of the network. The method estimates sparse symptom networks by shrinking weak connections to zero, reducing false positives and improving interpretability. It handles high-dimensional data, controls for multicollinearity, reveals direct associations, and allows tunable sparsity.^13^ Model selection was performed with the extended Bayesian information criterion (EBIC) that extends BIC by adding a penalty term that discourages overfitting, making it more suitable for high-dimensional data.^14^ A network comprising only significant edges was then generated, and clusters of correlated symptoms were grouped and assigned new variable names, referred to as “syndromes”. The networks were analysed and visualised via the R packages EstimateGroupNetwork and qgraph.^12^

### Between-group comparisons

Between-group differences in demographic and medical history data, as well as in the results of the various scales, were assessed using chi-square or Fisher’s exact tests for qualitative variables, and Kruskal–Wallis tests or t-tests for quantitative variables.

Linear models, adjusted for age, sex assigned at birth, and body mass index (BMI), were used to compare biomarker concentrations between the PASC and Recovered groups. Biomarker values were log-transformed and standardised, allowing them to be expressed in units of standard deviation (see equations in Supplement 1 p5). In this context, the estimate refers to the effect size derived from the statistical model, reflecting both the magnitude and direction of the association between biomarker variation and the group comparisons of interest. A first model comparing PASC and recovered with an additional adjustment for the duration since infection was developed. Then, two models focusing on subgroups determined by a confirmed infection less vs. more than one year ago were developed.

Based on the assumption that the pathophysiological pathways may vary according to specific symptoms, we then examined the association between each symptom or syndrome cluster and biomarker levels. This was done by comparing participants with PASC who reported having each persistent symptom to two groups: participants who had recovered from SARS-CoV-2 infection, and participants with PASC who did not report the symptom in question. We applied the same GLM procedure as describe above, along with the same subgroup analyseses stratified by time since infection.

Adjusted contrast methods (Tukey Honestly Significant Difference test) were used to perform multiple comparisons between the following groups: PASC group with each symptom vs. PASC group without each symptom; PASC group with each symptom vs. recovered; and PASC group without each symptom vs. recovered. Considering the analysis by symptoms, subgroups may overlap. We defined different p-value thresholds using Bonferoni method, ranging from treating biomarker results as fully independent within each symptoms group (14 tests for each symptoms/syndromes; significant p-value < 0.0036) to assuming full dependency (378 tests; p < 0.00013).

A sensitivity analysis was performed for participants whose symptoms were attributed to SARS-CoV-2 infection by a physician using the same model as above.

### Analysing M6 biomarkers and comparing M0 and M6

To further explore associations between PASC status and biomarkers, we compared participants who experienced complete resolution of PASC symptoms by M6 (Recovered-PASC) with those who continued to report symptoms at M6 (Persistent-PASC). This comparison was also performed at the individual symptom level — that is, participants with persistence of a specific symptom at M6 were compared with those who reported its resolution. Both absolute biomarker values at M6 and biomarker trajectories from M0 to M6 were analysed using linear regression models, adjusted for the same covariates as described above and using the same significant threshold.

All the statistical analyses were performed via R 4.2.1 software (Vienne).

### Role of the funding source

This study was funded by the French Ministry of Health and Prevention and the French Ministry of Higher Education, Research and Innovation. They had no role in the design, the data collection and analysis.

### Ethic statement

This research has been registered with the French authorities (IDRCB: 2020-A01195-34). Ethics committee approval was obtained according to the regulations in France (“comité de protection des personnes Sud Méditerranée III aproval number: 2021.09.08 cinq_ 21.02744.000038). Written informed consent was obtained from all participants.

## Results

### Participants

Among the 1000 participants enrolled in the COPER cohort between June and November 2022, 35 did not have completed data, 36 had a SARS-CoV-2 infection during the 3 months preceding the M0 visit and 128 had no known history of SARS-CoV-2 infection. Overall, 801 participants with confirmed history of SARS-CoV-2 infection were included in the study and classified into two groups: recovered (n = 490) and PASC (n = 311) (Fig. 1). Medical history before the study is shown in Supplementary Table S2, Appendix 1 p6. Participants with PASC were more likely to be women (250/311 (80.4) vs. 339/490 (69.2), p = 0.001 [χ^2^ test]), had a higher BMI (25.1 vs. 23.9, p < 0.001 [χ^2^ test]), and were infected earlier during the pandemic than those who recovered. Description of participants by sex is provided Supplementary Table S3, Appendix 1 p7.

### Clinical characterization of PASC

Within the PASC group, the median number of persistent symptoms was 3 [IQR: 1–9.5.5]. The most frequent symptoms were asthenia, dyspnoea, cough, and sleep disorders (Supplementary Table S4, Appendix 1 p8). Depression and anxiety symptoms were more prevalent in the PASC group as well (Table 1).

**Table 1:** Scale defining symptoms/syndromes and/or their intensity at the first visit.

Scales results	Recovered, n (%) (n = 490)	PASC, n (%) (n = 311)	p
Sex at birth			0.001^a^
Female	339 (69.2)	250 (80.4)	
Male	151 (39.8)	61 (19.6)	
Dyspnoea grade (MRC2 scale)			<0.001^a^
0	381 (77.8)	142 (45.8)	–
1	104 (21.2)	139 (44.8)	–
2	5 (1.02)	28 (9.0)	–
3	0 (0)	1 (0.3)	–
Depression scale (CES-D scale)			<0.001^a^
Depression	49 (10.3)	99 (33.2)	–
No depression	428 (89.7)	199 (66.8)	–
Anxiety scale (GAD-7 scale)			0.030^a^
Mild anxiety	30 (6.12)	33 (10.6)	–
No anxiety	460 (93.9)	278 (89.4)	–
Nijmegen score (>22.1)	71 (18.1)	86 (49.7)	<0.001^a^
Cognitive scale (MoCA)	27.0 [25.0; 28.0]	26.0 [24.0; 27.0]	<0.001^b^
Fatigue (MFI scale)			–
General	10.0 [7.00; 13.0]	13.0 [11.0; 15.0]	<0.001^b^
Physical	10.0 [7.00; 12.0]	11.0 [10.0; 13.0]	<0.001^b^
Mental	8.00 [4.00; 12.0]	10.0 [6.00; 13.0]	<0.001^b^
Activity	8.00 [5.00; 11.0]	10.0 [7.00; 13.0]	<0.001^b^
Motricity	7.00 [4.00; 11.0]	10.0 [7.00; 12.0]	<0.001^b^
Overall	46.0 [33.0; 57.0]	54.0 [46.0; 63.0]	<0.001^b^

a p calculated by: χ^2^ test.

b p calculated by: t-test.

**Fig. 2:**
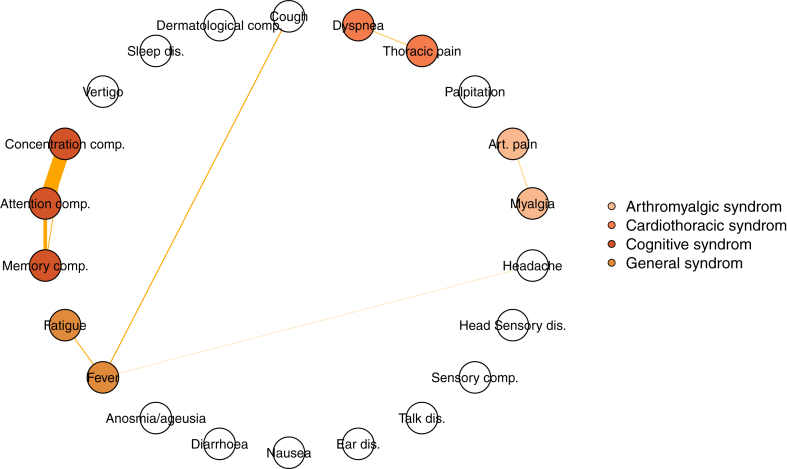
**Network analysis of persistent symptoms in participants with PASC.** The thickness of the link indicates the strength of the association. Symptoms involved in syndrome are coloured. comp: complaints; dis: disorder, art: articular.

Pearson correlation analysis revealed weak correlations between symptoms (Supplementary Table S6 and Supplementary Fig. S1, Appendix 1 p 10–11). Moreover, network analyses revealed very few significant associations between symptoms (Fig. 2). We identified four categories of syndromes: a ‘thoracic’ syndrome (defined by the association of dyspnoea and thoracic pain), a ‘cognitive’ syndrome (defined by the association of the presence of attention, concentration and memory complaints), a ‘general’ syndrome (defined by the association of fever and asthenia), and an ‘arthromyalgic’syndrome (association of myalgia and arthralgia).

### Associations between biomarkers, symptoms, and syndromes at M0

#### Comparison of PASC to recovered: impact of PASC status

In the first step of the analysis, we compared the entire PASC group without considering individual symptoms. No significant association was found between any of the biomarkers and PASC status (Supplementary Fig. S2, Appendix 1 p 12). Significant thresholds tended to be reached for PD-L1 (decreased in PASC). We then considered time elapsed since the last infection. Among participants with PASC who had been infected less than a year prior M0, significant thresholds tended to be reached for ICAM-1 (increased in PASC), and FABP2 (decreased in PASC). These differences were not observed among participants with PASC who had been infected more than a year earlier. For individuals with PASC infected more than a year ago significant threshold tended to be reached for PD-L1 (decrease in PASC) (Supplementary Fig. S2, Appendix 1 p 12).

#### Comparison of PASC to recovered: impact of the different PASC symptoms

We then hypothesised that PASC may be associated with distinct biomarker profiles depending on the symptoms present. To explore this, we analysed associations between individual symptoms and biomarkers by comparing all participants with PASC who reported a specific symptom to those in the Recovered group. Few symptoms were linked to biomarkers, and the observed associations were generally weak (Supplementary Fig. S3, Appendix 1, p13). We then examined these associations within subgroups defined by the time since infection. A greater number of biomarkers were associated with specific symptoms among participants infected less than one year prior to M0, compared to those infected more than one year before. Inflammatory biomarkers associated with COVID-19 severity (IFNγ, and CD163) were associated with some symptoms and syndromes. Biomarkers associated with vascular damage (VCAM-1, ICAM-1) were also associated with several symptoms and syndromes. Additionally, certain markers of viral activation (PD-L1, IP-10) appeared to be positively associated with participants symptoms that are associated with the acute phase of COVID-19: anosmia/ageusia and cough. These two symptoms were also the ones associated with the greatest number of biomarkers (Table 2a). When applying the more conservative p-value threshold, significant associations between biomarkers and symptoms persisted only for anosmia/ageusia. As the infection had occurred more than a year prior to baseline (M0) for most participants, fewer symptoms or syndromes remained associated with biomarkers, and none demonstrated strong associations when applying the more stringent significance threshold (Table 2b) (See Supplementary Figs. S4 and S5, Appendix 1 p14-15 for the full results).

**Table 2:** Association between each persistent symptom/syndrome and biomarker level when comparing participant with PASC with each symptom/syndrome to recovered participants.

a) PASC with S. vs. recovered: subgroups: less than one year before M0'			
Molecules	Symptoms/syndromes	Estimate (95% CI)	p value
IFNg	Cough	0.56 (0.07, 1.05)	0.04312
	Anosmia/ageusia	1.68 (0.95, 2.41)	**0.00002**
IL-6	Nausea	2.56 (0.60, 4.52)	0.02345
TNFa	Thoracic syndr.	2.82 (0.80, 4.84)	0.01523
IL-8	Memory comp.	0.76 (0.07, 1.45)	0.04843
IP-10	Cough	0.74 (0.25, 1.23)	0.0051
	Anosmia/ageusia	2.06 (1.33, 2.79)	**<0.00001**
PD-L1	Anosmia/ageusia	1.26 (0.44, 2.08)	0.00969
VCAM-1	Ear dis.	−1.04 (−1.88, −0.20)	0.02548
ICAM-1	Cognitive syndr.	1.14 (0.26, 2.02)	0.03755
	Cough	0.75 (0.30, 1.20)	0.00466
	Sensory comp.	1.17 (0.27, 2.07)	0.03747
	Anosmia/ageusia	1.38 (0.65, 2.11)	**0.00092**
	Memory comp.	0.94 (0.33, 1.55)	0.00898
CD163	Cognitive syndr.	1.19 (0.27, 2.11)	0.03757
	Anosmia/ageusia	1.15 (0.39, 1.91)	0.01099

b) PASC with S. vs. recovered: subgroups: more than one year before M0			
Molecules	Symptoms/syndromes	Estimate (95% CI)	p value
IFNg	Talk dis.	0.81 (0.19, 1.43)	0.01552
IL-8	Sensory comp.	−0.51 (−0.9, −0.11)	0.03427
IL-18	Dermatological comp.	−0.52 (−0.97, −0.06)	0.04778
PD-L1	Memory comp.	−0.39 (−0.64, −0.13)	0.01006
	Concentration comp.	−0.28 (−0.52, −0.05)	0.03019
ICAM-1	Diarrhoea	0.49 (0.08, 0.89)	0.02752
FABP	Talk dis.	0.82 (0.2, 1.43)	0.01379
	Vertigo	−0.48 (−0.88, −0.07)	0.04491

Results of the multivariable analysis adjusted on age, sex, and BMI in participants whose blood samples and SARS-CoV-2 infection occurred less than one year ago (a) or more than one year before the study (b). Syndromes are those defined by the network model analysis. Significant values (p < 0.05) are shown and p-value under 0.00013 are in bold.

comp: complaints; dis: disorder; hypervent. synd.: suspected hyperventilation syndrome (Nijmegen score above 22). Syndr.: Syndromes.

#### Comparison within PASC groups: impact of PASC symptom

We then considered that, within the PASC group, biomarker levels might vary depending on the presence or absence of specific symptoms. Largely, the same biomarkers were found to be associated with the same symptoms among participants infected less than one year prior to M0. Anosmia/ageusia remain the main symptom associated with the more biomarkers with the more strength (IFNγ, IP-10, ICAM-1) (Table 3a). In the subgroups of participants infected more than a year before M0, strength of association were less significant despite the highest number of individuals in this subgroup (Table 3b and Supplementary Figs. S3–S5, Appendix 1 p13-15).

**Table 3:** Associations between each persistent symptom/syndrome and biomarker level when comparing participant with PASC with each symptom/syndrome to those without the symptom/syndrome.

a) PASC with S. vs. PASC without S. : subgroups: infection less than a year before M0			
Biomarkers	Symptoms/syndromes	Estimate (95% CI)	p value
IFNg	Cough	0.77 (0.16, 1.37)	0.043
	Anosmia/ageusia	1.79 (1.02, 2.55)	**0.00002**
IL-6	Nausea	2.46 (0.49, 4.43)	0.02344
TNFa	Thoracic syndr.	2.71 (0.67, 4.75)	0.01523
IL-8	Memory comp.	0.85 (0.11, 1.59)	0.04843
IP-10	Cough	0.89 (0.31, 1.48)	0.00510
	Anosmia/ageusia	2.11 (1.35, 2.87)	**<0.00001**
PD-L1	Anosmia/ageusia	1.13 (0.27, 2.0)	0.01659
VCAM-1	Ear dis.	−1.17 (−2.04, −0.3)	0.02548
ICAM-1	Cough	0.74 (0.19, 1.3)	0.01418
	Ear dis.	−1.0 (−1.84, −0.16)	0.03125
	Anosmia/ageusia	1.21 (0.45, 1.96)	0.00343
CD163	Cognitive syndr.	1.1 (0.14, 2.05)	0.03851
	Anosmia/ageusia	1.11 (0.31, 1.91)	0.01099

b) PASC with S. vs. PASC without S. : subgroups: infection more than a year before M0			
Biomarkers	Symptoms/syndromes	Estimate (95% CI)	p value
IFNg	Talk dis.	0.92 (0.31, 1.54)	0.01070
IL-6	Art. pain	−0.39 (−0.71, −0.08)	0.04531
	Sensory comp.	−0.52 (−0.9, −0.13)	0.02810
IL-8	Sensory comp.	−0.46 (−0.85, −0.06)	0.03427
IL-18	Dermatological comp.	−0.51 (−0.97, −0.04)	0.04778
IP-10	Fatigue	0.32 (0.09, 0.55)	0.01224
VCAM-1	Cough	0.37 (0.05, 0.68)	0.03354
	Memory comp.	0.3 (0.03, 0.57)	0.04868
ICAM-1	Diarrhoea	0.62 (0.22, 1.03)	0.00787
	Vertigo	0.5 (0.1, 0.9)	0.046737
FABP	Talk dis.	0.91 (0.29, 1.53)	0.01168
	Attention comp.	0.32 (0.06, 0.58)	0.04616
	Vertigo	−0.45 (−0.86, −0.04)	0.04491

Results of the multivariable analysis adjusted on age, sex, and BMI in participants whose blood samples and SARS-CoV-2 infection occurred less than one year ago (a) or more than one year before the study (b). Syndromes are those defined by the network model analysis. Significant values (p < 0.05) are shown and p-value under 0.00013 are in bold.

comp: complaints; dis: disorder; hypervent. synd.: suspected hyperventilation syndrome (Nijmegen score above 22). Syndr.: Syndromes.

#### Analysis concerning participants with medically-validated-PASC: sensitivity analysis

Participants with PASC who had at least one persistent symptom deemed by a physician to be associated with SARS-CoV-2 infection comprised one-third of the overall PASC group (medically validated-PASC, n = 102) (see Supplementary Table S5, Appendix 1 p 9). We confirmed weak associations between biomarkers and symptoms considering all the medically-validated-PASC and in those with infection having occurred more than one year ago (Supplementary Fig. S6, Appendix 1 p16). In participants with medically-validated-PASC infected less than one year ago, we confirmed the associations of acute COVID-19 with biomarkers that may be associated with severity, viral activation and vascular damage/activation ((IFNγ, IP-10, PD-L1, VCAM-1, ICAM-1)) with some symptoms (Supplementary Figs. S7–S9, Appendix 1 p17-19). Anosmia/ageusia remained the symptom the more associated with these biomarkers.

#### Evolution of symptoms and biomarkers between M0 and M6

**Fig. 3:**
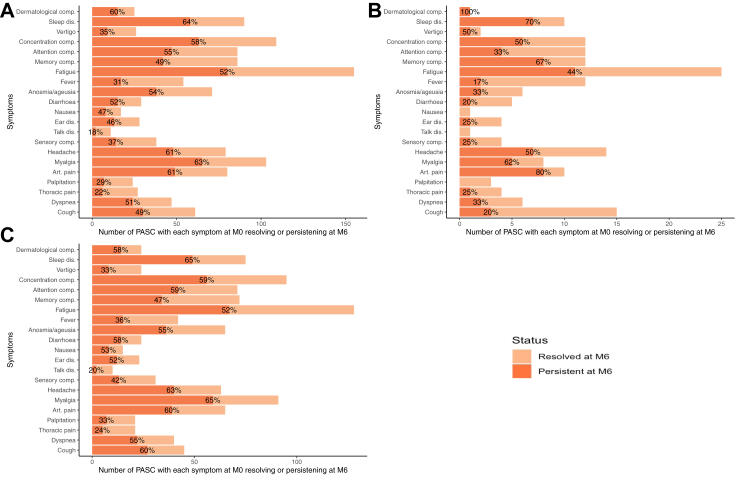
**Resolution of symptoms between M0 and M6 among the participants with PASC.** A) Overall PASC; B) PASC infected less than one year before M0; C) PASC infected more than one year before M0. Proportion of persistent symptoms at M6 are shown in percentage. comp: complaints; dis: disorder.

At M6, 748/929 (81%) of the respondents completed the survey and had available sera, including 251/311 (81%) with PASC at M0, 392/490 (80%) without PASC. Among participants with PASC at M0, 55/251 (22%) experienced complete resolution of their symptoms (Recovered-PASC). Complete resolution was more frequent in those with a more recent infection, with 12/32 (38%) and 43/219 (20%) (p = 0.04 [Fisher's exact test]) recovery in the groups defined by an infection less than a year ago at the first visit and more than a year ago at the first visit, respectively. At least one symptom was resolved in 185/251 (74%) individuals. In the 196 participants with no or partial symptom resolution, the median number of symptoms was 2 [IQR 1–4]. The number and the proportion of participants who recovered and who do not recovered from each symptom is shown in Fig. 3 and Supplementary Fig. S10, Appendix 1 p20.

Value of the biomarkers at M6 between Recovered-PASC and Persistent-PASC were not different. We then compared the variation of concentration of each biomarker between M0 and M6 between the recovered-PASC and the persistent-PASC groups (Supplementary Fig. S11, Appendix 1 p 21). IP-10 was the only biomarker weakly associated with recovery status in the full model, and IFNγ was weakly associated with PASC resolution in those with a more recent infection. The most conservative significance threshold remained unmet.

We then compared participants reporting a particular symptom to those in whom the symptom had resolved. Given the sample sizes, it was not possible to study all symptoms disappearance in all subgroups, especially among those infected less than one year before M0. Considering the association between biomarker levels at M6 (Supplementary Table S7a, Supplement 1, p. 22) and the variation in biomarker levels between M0 and M6 (Supplementary Table S7b, Supplement 1, p. 22), the significance threshold tended to be reached for only a few symptoms, typically with a single biomarker. However, the most conservative significance threshold remained unmet. The resolution of anosmia/ageusia and fatigue showed the strongest associations with changes in biomarker levels, suggesting a closer link between these specific symptoms and underlying biological processes.

## Discussion

In this study, we observed variations in the biomarker profile between participants with PASC and those who had recovered from SARS-CoV-2 infection. These differences were more strongly associated with certain symptoms than others and were less evident in participants infected more than a year before the study, despite these individuals reporting a higher number of symptoms.

In this well-characterised cohort drawn from the general population, we were able to investigate dependencies among symptoms using a network-based approach. In pathophysiological research, this method may help to identify symptom clusters with shared underlying mechanisms, thereby facilitating biomarker discovery. Some symptoms did not seem to be associated with others, notably anosmia/ageusia, as previously suggested.^15^ The weak associations observed between symptoms suggest that distinct underlying mechanisms may drive their onset and persistence. This interpretation is supported by the absence of strong biomarker signals in the overall PASC group and the variability in associations across individual symptoms. It is therefore essential to avoid treating PASC as a homogeneous condition. Future research must carefully characterise individuals based on their symptom profiles to ensure reproducibility and to avoid misleading attributions. For example, recent studies on PASC often included many participants with anosmia/ageusia, linking this symptom to neurological disorders, highlighting the need for a symptom-specific approach in biomarker and pathophysiological investigations.^16^^,^^17^

Symptom-based analyseses revealed associations between certain symptoms and biomarkers linked to viral activation, including IFNγ, PD-L1, and IP-10. This is in line with recent literature suggesting that viral persistence may contribute to the persistence of symptoms. However, the detection of viral particles in tissues and/or blood has not been consistently observed across all studies.^18^^,^^19^ This may once again be explained by the heterogeneity of the patient population studied. In our cohort, we conducted complementary analyses to investigate the presence of SARS-CoV-2 antigen (spike protein) and found no differences between patients with PASC and those who recovered (data not shown). Furthermore, the link between this viral persistence and the continuation of symptoms appears to diminish over time.^20^ This is consistent with our findings that the association between PASC and biomarkers were predominantly observed in the subgroup with more recent infection, despite these participants reporting fewer symptoms than those infected over a year earlier. This may suggests dynamic changes in the underlying pathophysiology over time.^21^ The longitudinal analysis further support this assumption. Several, non-exclusive explanations may account for these observations: 1) Persistent symptoms may become independent of the initial process over time, as observed in many medical conditions; 2) Immunological exhaustion may make those markers in peripheral blood less easily detectable over time while the mechanisms may persist within tissues; 3) Other mechanisms associated with the intensity of the acute phase may be associated with symptoms such as autoimmune reactions or nonspecific processes.^22^ These findings have direct implications for the therapeutic approach to PASC, which should take symptomatology into account, as the underlying mechanisms may differ. This suggest a need for tailored treatments that also consider the time elapsed since infection. For example, one could hypothesise that the absence or disappearance of biomarkers associated with viral replication could predict reduced efficacy of treatments targeting this mechanism.

We identified associations between certain symptoms and biomarkers linked to severity, such as IL-8 and CD163, which are involved in multiorgan dysfunction and infection resolution, particularly in recent infections.^23^^,^^24^ These findings suggest that inflammatory biomarkers may aid in diagnosing PASC in its early phase and in assessing the risk symptom persistence. Targeting these inflammatory pathways may be most effective early in the disease course. Finally, biomarkers of endothelial activation, including VCAM-1 and ICAM-1, tended to be associated with cognitive symptoms. Vascular damage and endothelial activation have previously been proposed as mechanisms underlying COVID-19 severity, as well as certain pulmonary and cognitive manifestations.^25^^,^^26^ Our findings also support this hypothesis but require further investigation, including the identification of more specific biomarkers and their correlation with biomarkers of cerebral distress.

The strength of this work lies in the large number of participants with PASC, which had no history of hospitalisation for COVID-19. Similar data are scarce and include some hospitalised participants most of the time.^27^, ^28^, ^29^ The size of the cohort led to the possibility of subgroup analysis and statistical adjustments for variables known to be associated with the severity of COVID-19, and/or those associated with variations in biomarkers values (age, sex, BMI).

This study has several limitations. Our definition of PASC differs from that of the WHO; however, sensitivity analysese restricted to cases attributed to SARS-CoV-2 by a physician yielded similar results, supporting the validity of the sample. Selection bias is possible, as severely affected individuals may have been more likely to participate. However, symptom prevalence and distribution align with general population data, and the male-to-female ratio reflects PASC’s higher prevalence in women.^4^ Most PASC data derive from 2020 to 2021, making biomarker collection distant from infection. Biomarker differences largely disappear within a year, and the small group with recent infections may have limited detection of acute-phase associations.^30^ Finally, the decision to focus on a limited number of biomarkers in this initial analysis was guided by the rationale of prioritising key pathophysiological pathways that have been partially explored to date, and often with inconsistent findings. Broader investigations incorporating high-throughput approaches such as proteomics are needed to comprehensively elucidate the underlying mechanisms.

In conclusion, our findings confirmed that individuals with PASC do not represent a homogeneous pathophysiological group. Biomarker profiles appear to vary according to symptom type and the time elapsed since infection. Consequently, research efforts and treatment strategies should take these parameters into account.

## Contributors

OR and FC conceived of the present idea. OR, SH, CD, JN, JDL, MZ, EW and FC wrote the protocol. EW, CR, MG, GS, MT, MZ implemented the study within the three cohorts. HB and JFD were in charge of the sample storage. SH, MS and SB did the biological analysis. OR and FC conceived the data analysis, had full access to the data, and verified the underlying data. OR, SH, XDL, JDL and FC discuss the results. OR wrote the first proof of the manuscript with support from SH, CL, YL, XDL, JDL and FC. All authors discussed the results and commented on the manuscript. All authors read and approved the final version of the manuscript.

## Data sharing statement

The dataset used for this study is third-party data and not publicly available to ensure the confidentiality of study participants sensitive data. The data are already accessible upon approval by the COPER scientific committee based on a written project submitted by research teams. Their release will require approval from the relevant French authorities and a data sharing agreement between the institution hosting the data (INSERM) and the requesting laboratory. Colleagues interested in working on our datasets are encouraged to contact Pr Olivier Robineau (olivier.robineau@univ-lille.fr).

## Declaration of interests

OR declare financial support from Gilead, ViiV, MSD, Moderna, Pfizer outside the submitted work. CL reports non-financial support from Nordic Pharma France, outside the submitted work. Other authors have nothing to declare.
