# Supplementary material for: Symptoms and pathophysiology of post-acute sequelae following COVID-19 (PASC): a cohort study
Source: eBioMedicine. 2025 May 30;117:105792. doi: 10.1016/j.ebiom.2025.105792 (PMC12166779; doi:10.1016/j.ebiom.2025.105792)
Supplement: Supplementary Figs. S1–S11 and Tables S1–S7 [file mmc1.docx]

**Supplementary materials**

**Table of contents**

[Participants selection 2](#_Toc193666372)

[Sample processing 4](#_Toc193666373)

[Serological analysis of individual without known history of SARS-CoV-2 infection 4](#_Toc193666374)

[Table S1 biomarkers analysed. 5](#_Toc193666375)

[Statistical analysis: model used 5](#_Toc193666376)

[Table S2 Characteristics of the participants. 6](#_Toc193666377)

[Table S3 Description of the population by sex. 7](#_Toc193666378)

[Table S4 Clinical characteristics and persistent symptoms among participants with PASC by time from infection. 8](#_Toc193666379)

[Table S5 Description of the population according to whether the symptoms were confirmed by a doctor (*medically-validated-PASC*) or not (*Unconfirmed* PASC). 9](#_Toc193666380)

[Table S6 Correlation matrix of symptoms. 10](#_Toc193666381)

[Figure S1 Correlation analysis of persistent symptoms in PASC. The thickness of the link indicates the strength of the association. Symptoms usually associated to a system are of the same colour. 11](#_Toc193666382)

[Figure S2 Association between PASC status and biomarker levels: Results of the multivariable analysis of the overall population comparing participants with PASC and those who recovered; subgroup analysis by the time from infection. 12](#_Toc193666383)

[Figure S3 Associations between each persistent symptom and biomarker levels: Results of the multivariable analysis of the overall population adjusted for time from infection. 13](#_Toc193666384)

[Figure S4 Associations between each persistent symptom and biomarker levels: Results of the multivariable analysis of the participants with the last infection having occurred less than one year before the study. 14](#_Toc193666385)

[Figure S5 Associations between each persistent symptom and biomarker levels: Results of the multivariable analysis of the participants with the last infection having occurred more than one year before the study. 15](#_Toc193666386)

[Figure S6 Association between medically-confirmed-PASC and biomarker levels: Results of the multivariable analysis of the overall population; subgroup analysis by the time from infection. 16](#_Toc193666387)

[Figure S7 Association between each persistent symptom in the medically-confirmed-PASC subgroup and biomarker level: Results of the multivariable analysis of the overall population. 17](#_Toc193666388)

[Figure S8 Association between each persistent symptom in the subgroup of medically-confirmed-PASC and biomarker level: Results of the multivariable analysis of participants whose time between blood sample collection and SARS-CoV-2 infection was longer than one year. 18](#_Toc193666389)

[Figure S9 Association between each persistent symptom in the subgroup of medically-confirmed-PASC and biomarker level: Results of the multivariable analysis of participants whose time between blood sample and SARS-CoV-2 infection was less than one year. 19](#_Toc193666390)

[Figure S10 Proportion of resolution of symptoms between M0 and M6 among participants with PASC depending on time from infection: A) All PASC; B) PASC with infection lasting less than one year prior to M0; C) PASC with infection lasting more than one year prior to M0. Proportion of persistent symptoms at M6 are shown in percentage. comp: complaints; dis: disorder. 20](#_Toc193666391)

[Figure S11 Variation in biomarkers among Recovered-PASC participants at M6 and persistent PASC participants at M6. Significant values are in bold. 21](#_Toc193666392)

[Table S7 Association between biomarkers and symptoms resolution 22](#_Toc193666393)

### Participants selection

This study was conducted using data from the SAPRIS-Sero multi-cohort study.^1^ Briefly, the SAPRIS-Sero cohort is based on a consortium of prospective cohort studies (Constances, E3NE4N and Nutrinet-Santé) in the general population including 279,478 adult volunteers with regular access to electronic (internet) questionnaires. Two self-administered questionnaires covering the first lockdown and the post-lockdown periods were sent in April and May 2020. The questionnaires included socio-demographics, history of COVID-19 diagnosis and SARS-CoV-2 RT-PCR testing, a detailed description of the participant’s symptoms in the previous weeks, and an invitation to perform a serology by self-sampling dried-blood spot (DBS). A total of 77,580 of these participants had a serology result and had completed the 2 questionnaires. A follow-up questionnaire was then sent between June and October 2021 in those participants (N=56 604). It included detailed information on vaccination, diagnosis of SARS-CoV-2 infection, symptoms and healthcare use since the 2020 questionnaires.^2^ A total of 53,047 participants had complete data and were selected to be eligible for the COPER cohort.

On the basis of these questionnaires and the serological results, we were able to identify participants infected with SARS-CoV-2 between February 2020 and October 2021, defining *infection* as documentation of a positive RT-PCR over this period, or the existence of positive serology on the first serological sample following the first 2020 pandemic wave (prior to any vaccination) ; and *persistent symptoms*, symptoms lasting at least two months that appeared within one month of infection and still present at the time of the most recent questionnaire.

Four groups of participants were thus *a priori* defined:

- participants who did not report a SARS-CoV-2 infection and had no history of positive serology for SARS-CoV-2, defining the group with "no known history of SARS-CoV-2 infection"
- participants who had a confirmed SARS-CoV-2 infection but remained asymptomatic during the acute phase, defining the "asymptomatic SARS-CoV-2 infection - recovered " group
- participants who had a confirmed SARS-CoV-2 infection and experienced symptoms during the acute phase without persistent symptoms, defining the "symptomatic SARS-CoV-2 infection - recovered" group
- and participants who had a confirmed SARS-CoV-2 infection and had persistent symptoms following a symptomatic acute infection, defining the "PASC" group.

598 participants of SAPRIS-Sero were defined as PASC. Initially, as specified in the COPER cohort protocol, we aimed to include three hundred participants in each group with individual matching on age (+/-5 years), gender, and cohort, based on the PASC group. Eligible participants were invited to join the COPER cohort, between June 2022 and November 2022 (2022 inclusion questionnaire). PASC were randomly selected and matched to other groups. We maintained a possibility to select new participants during the inclusion period if the objective of inclusion were not reached. During the 6-month inclusion period, 1,000 people received a home visit from a research nurse to give written consent to participate in the study, complete hetero-questionnaires, and have biological samples taken. More PASC participants than expected were included because the acceptance rate was higher than initially estimated. The other groups had a lower acceptance (See figure S1).

For the research work present in this paper, We did a selection process using the 2022 inclusion questionnaire that excluded participants with a history of COVID-19 during the 3 months preceding the first visit, those who did not complete the questionnaire at the first visit of the COPER survey, and/or those whose samples could not be analysed.

Then, given the risk of new infection and/or favourable evolution of participants with PASC since the initial screening in 2021, some participants were reallocated to other group. We reclassified participants by considering the symptoms present at the time of the first visit in the COPER cohort:

- Participants initially considered with PASC based on the online questionnaires but no longer symptomatic at the time of the 2022 inclusion questionnaire were re-classified in this 'Recovered' group (n=69)
- Participants initially selected as having "no known history of SARS-CoV-2 infection" and did not declare a SARS-CoV-2 infection between the 2022 questionnaire and the first visit underwent new serological tests, and were also reclassified into the “Recovered” group in case of positive serological results (n=24).
- From any intial groups, participants that declare a SARS-CoV-2 infection between the 2022 questionnaire and the visit may be allocated to the “recovered” group or the PASC group depending on the symptoms at the first visit in the COPER cohort.

Due to significant imbalances and the reclassification of participants into different groups after inclusion in the study, the statistical analysis does not take into account the matching that was done a priori. Thus, model were adjusted consequently when possible (see the method section in the article).

Ultimately, our objective was to compare participants with a history of SARS-CoV-2 infection with no symptoms at the first visit to those who still had symptoms at the time of the study. Therefore, we defined the 'Recovered' group as all participants who had previously been infected with SARS-CoV-2 and were symptom-free at the visit and the PASC group as all participant with at least persistent symptom.

### Sample processing

Serum samples were collected from the participants in Vacutainer tubes and kept at room temperature for 30 to 45 minutes after the blood was drawn to assess clot formation prior to centrifugation at 2000 × g at 4°C for 10 minutes. The samples were aliquoted 0.25 or 0.50 mL into 1.5 mL polypropylene tubes and stored in -80°C freezers equipped with a 24-hour alarm system for detecting temperature excursions triggering an alert sent to on-call personnel.

### Serological analysis of individual without known history of SARS-CoV-2 infection

SARS-CoV-2 nucleocapsid and spike-specific IgG antibodies were detected using the Meso Scale Discovery (MSD) platform, V-PLEX SARS-CoV-2 Key Variant Spike Panel 1 Kit (catalog number K15651). Briefly, serum samples were diluted 1:5000 prior to the assay. Kit plates were blocked for 30 min using manufacturer-provided blocking buffer A. Blocking buffer was removed, and plates were washed 3X with wash buffer. Diluted serum samples, controls, and calibrators were added to the plates and incubated for 2 h at room temperature in a plate shaker adjusted at 700 rpm. After a 2-h incubation, plates were washed 3X, loaded with a solution containing MSD Sulfo-Tag anti-human IgG, and incubated for 1 h in a plate shaker adjusted at 700 rpm. After incubation, the plates were washed 3X, and the detection reading buffer was added immediately prior to the plate read. Antibody unit concentrations (AU/ml) were determined from their ECL signals by backfitting to the calibration curve established by fitting the signals from the calibrators to a 4-parameter logistic model with a 1/Y2 weighting. Positivity thresholds were set as the mean concentration + 3xSD (or 99th percentile) of 74 pre-COVID healthy donor samples: 1502 (or 1865) AU/ml for anti-Spike and 8255 (or 9569) AU/ml for anti-Nucleocapsid IgG.

This procedure was performed among sample of individual with no history of positive PCR or serology. Individual positive for anti-Nucleocapsid IgG were considered as recovered from SARS-Cov-2 infection (n=34).

###

### Table S1 biomarkers analysed.

| **Biomarkers** | **Description** |
| --- | --- |
| **IFNγ** | Interferon-gamma: Cytokine involved in immune response, macrophage activation, and immune cell regulation |
| **IL-1b** | Interleukin-1 beta: Pro-inflammatory cytokine regulating immune and inflammatory responses |
| **IL-6** | Interleukin-6: Cytokine with various functions, including immune response regulation and inflammation |
| **IL-8** | Interleukin-8: Chemokine stimulating immune cell migration to sites of infection or inflammation |
| **IL-18** | Interleukin-18: Cytokine involved in immune response regulation, particularly in natural killer cell activation |
| **IP-10** | Interferon-gamma-Induced Protein 10: Chemokine induced by interferon-gamma, playing a role in immune cell migration |
| **PD-L1** | Programmed Death-Ligand 1: Protein involved in immune regulation (viral infection, cancer) |
| **PDGF-BB** | Platelet-Derived Growth Factor BB: Growth factor involved in cell proliferation and wound healing |
| **TNFα** | Tumour Necrosis Factor-alpha: Pro-inflammatory cytokine regulating immune response and inflammation |
| **TRAIL** | Tumour Necrosis Factor-Related Apoptosis-inducing Ligand: Protein inducing apoptosis in certain cells |
| **VCAM-1** | Vascular Cell Adhesion Molecule 1: Cell adhesion molecule facilitating immune cell adhesion to blood vessel walls |
| **FABP2** | Fatty Acid-Binding Protein: Protein involved in the transport and metabolism of fatty acids |
| **CD163** | Scavenger receptor expressed on macrophages, involved in the clearance of haemoglobin-haptoglobin complexes |
| **ICAM-1** | Intercellular Adhesion Molecule 1: Cell adhesion molecule facilitating immune cell adhesion and migration |

### Statistical analysis: model used

To explore the association between biomarker levels and participant characteristics at M0, we fitted a linear model. The dependent variable was the biomarker level, which was normalized using a log transformation and then standardized (centered around the mean and scaled by the standard deviation). The independent variables included group status (PASC or symptom status), BMI category (BMIcal), sex, and age class (ageClass).

The model was specified as follows:BIomarker*_i_* =β_0_ + β_1_ group*_i_* + β_2_ BMIcal*_i_* + β_3_Sex*_i_*+β_4_ Ageclass*_i_* + ϵ*_i_*

where β_0_​ ​ represents the intercept, β_1_,β_2_,β_3_ ​, and β_4_​ ​ are the estimated coefficients for the respective categorical variables, and ϵi​ denotes the residual error term.

To assess the association between changes in biomarker levels over time and participant characteristics, we performed a linear regression analysis. The dependent variable was “intraIndiv”, representing the difference of biomarker levels between M0 and M6 which was normalized using a log transformation and then standardized (centered around the mean and scaled by the standard deviation) The independent variables included group status (PASC or symptom status), BMI category (BMIcal), sex, and age class (ageClass).

The model was specified as follows:

intraIndiv =β_1_+β_1_ Groups_i_ + β_2_ BMIcal*_i_* + β_3_ Sexe*_i_* +β_4_ Ageclass*_i_* +ϵ*_i_*

where β_0_​ represents the intercept, β_1_,β_2_,β_3_ ​, and β_4_​ are the estimated regression coefficients for each predictor variable, and ϵ_i_ ​ denotes the residual error term. All categorical variables were treated as factors.

### Table S2 Characteristics of the participants. (p calculated by ^1^: χ² test ; ^2^ : Fisher's exact test

### ; ^3^ : t-test)

| **Characteristics** | **Recovered, n (%) (n = 490)** | **PASC, n (%) (n = 311)** | **p** |
| --- | --- | --- | --- |
| **Cohorts involved in the study** |  |  | 0.039^1^ |
| Constance | 342 (64.3) | 190 (35.7) |  |
| E3N- Generations | 61 (54.5) | 51 (45.5) |  |
| Nutrinet | 87 (55.4) | 70 (44.6) |  |
| **Characteristics at the inclusion in the SAPRIS Survey** |  |  |  |
| Sex (female) | 339 (69.2) | 250 (80.4) | 0.001^1^ |
| Chronic respiratory disease | 26 (5.3) | 23 (7.4) | 0.293^1^ |
| History of anxiety or depression | 5 (1.0) | 12 (3.9) | 0.014^2^ |
| History of cancer | 11 (2.2) | 18 (5.8) | 0.015^2^ |
| Hypertension | 28 (5.7) | 20 (6.4) | 0.792^2^ |
| Diabetes mellitus | 9 (1.8) | 10 (3.2) | 0.312^2^ |
| Chronic cardiac disease | 12 (2.5) | 6 (1.9) | 0.811^2^ |
| Smoking status |  |  | 0.0391^2^ |
| Ex-smoker | 181 (36.9) | 135 (43.4) | ·· |
| Non-smoker | 247 (50.4) | 132 (42.4) | ·· |
| Smoker | 52 (10.6) | 39 (12.5) | ·· |
| **Characteristics at the first COPER visit** |  |  |  |
| Age, years | 50.0 [42.0; 60.8] | 51.0 [44.0; 60.0] | 0.333^3^ |
| BMI (kg/m^2^) |  |  | 0.003^1^ |
| < 25 | 332 (68.7) | 171 (56.6) | ·· |
| 25–30 | 109 (22.6) | 94 (31.1) | ·· |
| ≥ 30 | 42 (8.7) | 37 (12.3) | ·· |
| **Year of infection** |  |  | < 0.001^1^ |
| 2020 | 210 (42.9) | 160 (51.4) | ·· |
| 2021 | 140 (28.6) | 111 (35.7) | ·· |
| 2022 | 117 (23.9) | 40 (12.9) | ·· |
| Unknown | 23 (4.7) | 0 (0) | ·· |

### Table S3 Description of the population by sex. (p calculated by ^1^: t-test ; ^2^ : χ² test ; ^3^ : Fisher's exact test)

### t)

| Variables | Female N=686 | Male N=243 | p |
| --- | --- | --- | --- |
| age (mean) | 50.0 [43.0;60.0] | 55.0 [43.0;64.0] | 0.004^1^ |
| BMI (Kg/m2): |  |  | <0.001^2^ |
| <25 | 464 (69.4%) | 121 (50.4%) |  |
| 25-30 | 66 (9.87%) | 23 (9.58%) |  |
| >30 | 139 (20.8%) | 96 (40.0%) |  |
| Date of infection |  |  | 0.671^2^ |
| 2020 | 190 (42.5%) | 64 (43.0%) |  |
| 2021 | 165 (36.9%) | 50 (33.6%) |  |
| 2022 | 92 (20.6%) | 35 (23.5%) |  |
| **Symptoms** | | | |
| Hyperven. Syndr | 135 (29.7%) | 38 (17.9%) | 0.002^3^ |
| Cough | 101 (14.7%) | 41 (16.9%) | 0.486^3^ |
| Dyspnea | 55 (8.02%) | 22 (9.05%) | 0.713^3^ |
| Thoracic pain | 37 (5.39%) | 10 (4.12%) | 0.541^3^ |
| Palpitation | 55 (8.02%) | 8 (3.29%) | 0.018^3^ |
| Art. pain | 164 (23.9%) | 42 (17.3%) | 0.041^3^ |
| Myalgia | 186 (27.1%) | 68 (28.0%) | 0.859^3^ |
| Headache | 217 (31.6%) | 49 (20.2%) | 0.001^3^ |
| Head sensory comp. | 3 (0.44%) | 0 (0.00%) | 0.571^3^ |
| Sensory comp. | 84 (12.2%) | 14 (5.76%) | 0.007^3^ |
| Talk dis. | 20 (2.92%) | 6 (2.47%) | 0.892^3^ |
| Ear dis. | 62 (9.04%) | 10 (4.12%) | 0.020^3^ |
| Nausea | 51 (7.43%) | 5 (2.06%) | 0.004^3^ |
| Diarrhoea | 79 (11.5%) | 13 (5.35%) | 0.008^3^ |
| Anosmia/ageusia | 80 (11.7%) | 16 (6.58%) | 0.035^3^ |
| Fever | 70 (10.2%) | 20 (8.23%) | 0.443^3^ |
| Fatigue | 209 (30.5%) | 61 (25.1%) | 0.134^3^ |
| Memory comp. | 119 (17.3%) | 25 (10.3%) | 0.012^3^ |
| Attention comp. | 125 (18.2%) | 24 (9.88%) | 0.003^3^ |
| Concentration comp. | 152 (22.2%) | 35 (14.4%) | 0.013^3^ |
| Vertigo | 57 (8.31%) | 7 (2.88%) | 0.006^3^ |
| Sleep dis. | 230 (33.5%) | 47 (19.3%) | <0.001^3^ |
| Dermatological comp | 40 (5.83%) | 18 (7.41%) | 0.472^3^ |
| General synd. | 64 (9.33%) | 19 (7.82%) | 0.563^3^ |
| Neurological synd. | 76 (11.1%) | 13 (5.35%) | 0.013^3^ |
| Thoracic synd. | 4 (0.58%) | 1 (0.41%) | 1.000^3^ |
| Rheumatological syn. | 53 (7.73%) | 16 (6.58%) | 0.659^3^ |

### Table S4 Clinical characteristics and persistent symptoms among participants with PASC by time from infection (p calculated by ^1^: t-test ; ^2^ : Wilcoxon-Mann-Whitney Test ; ^3^ : Fisher's exact test)

|  | Time between SARS-CoV-2 infection and the survey | |  |
| --- | --- | --- | --- |
| Variables | Less than 1 year (N=41) | More than 1 year (N=270) | p |
| Age (mean) | 50.0 [45.0;59.0] | 52.0 [44.0;60.0] | 0.895^1^ |
| number of persistent symptoms (median [IQR] | 1 [1-4] | 3 [1-6] | .00539^2^ |
| BMI (kg/m2) |  |  | 0.419^3^ |
| <25 | 20 (50.0%) | 150 (57.9%) |  |
| 25-30 | 16 (40.0%) | 76 (29.3%) |  |
| >30 | 4 (10.0%) | 33 (12.7%) |  |
| **Symptoms** | | | |
| Cough | 20 (48.8%) | 66 (24.7%) | 0.003^3^ |
| Dyspnoea | 8 (19.5%) | 58 (21.7%) | 0.907^3^ |
| Thoracic pain | 5 (12.2%) | 29 (10.9%) | 0.790^3^ |
| Palpitation | 4 (9.76%) | 40 (15.0%) | 0.515^3^ |
| Articular pain | 9 (22.0%) | 108 (40.4%) | 0.036^3^ |
| Myalgia | 16 (39.0%) | 132 (49.4%) | 0.282^3^ |
| Headache | 24 (58.5%) | 123 (46.1%) | 0.187^3^ |
| Head sensory disorder | 1 (2.44%) | 1 (0.37%) | 0.249^3^ |
| Sensitive disorder | 7 (17.1%) | 50 (18.7%) | 0.970^3^ |
| Speech disorder | 3 (7.32%) | 17 (6.37%) | 0.738^3^ |
| Ear disorder | 6 (14.6%) | 39 (14.6%) | 1.000^3^ |
| Nausea | 2 (4.88%) | 26 (9.74%) | 0.557^3^ |
| diarrhoea | 10 (24.4%) | 41 (15.4%) | 0.221^3^ |
| Anosmia/ageusia | 6 (14.6%) | 85 (31.8%) | 0.039^3^ |
| Fever | 14 (34.1%) | 61 (22.8%) | 0.169^3^ |
| Asthenia | 26 (63.4%) | 175 (65.5%) | 0.928^3^ |
| Memory complaint | 10 (24.4%) | 99 (37.1%) | 0.160^3^ |
| Attention complaint | 10 (24.4%) | 99 (37.1%) | 0.160^3^ |
| Concentration complaint | 15 (36.6%) | 123 (46.1%) | 0.333^3^ |
| Vertigo | 2 (4.88%) | 40 (15.0%) | 0.131^3^ |
| Sleep disorder | 16 (39.0%) | 131 (49.1%) | 0.303^3^ |
| Dermatological disorder | 3 (7.32%) | 31 (11.6%) | 0.594^3^ |

### Table S5 Description of the population according to whether the symptoms were confirmed by a doctor (*medically-validated-PASC*) or not (*Unconfirmed* PASC). p calculated by ^1^: χ² test; ^2^ : t-test ; ^3^ : Fisher's exact test)

| Variables | *Unconfirmed* PASC  N=209 | *medically-validated-PASC*  N=102 | p |
| --- | --- | --- | --- |
| Sex |  |  | 0.170 ^1^ |
| Female | 163 (78.0%) | 87 (85.3%) |  |
| Male | 46 (22.0%) | 15 (14.7%) |  |
| age (mean) | 52.0 [44.0;61.0] | 51.0 [44.2;58.8] | 0.644^2^ |
| BMI (Kg/m2): |  |  | 0.227^1^ |
| <25 | 123 (60.0%) | 48 (49.5%) |  |
| 25-30 | 59 (28.8%) | 35 (36.1%) |  |
| >30 | 23 (11.2%) | 14 (14.4%) |  |
| Date of infection |  |  | 0.010^1^ |
| 2020 | 87 (48.1%) | 47 (51.6%) |  |
| 2021 | 60 (33.1%) | 39 (42.9%) |  |
| 2022 | 34 (18.8%) | 5 (5.49%) |  |
| **Symptoms** | | | |
| Hyperven. Syndr | 59 (47.2%) | 27 (56.2%) | 0.370^3^ |
| Cough | 59 (28.2%) | 27 (26.5%) | 0.849^3^ |
| Dyspnea | 31 (14.8%) | 35 (34.3%) | <0.001^3^ |
| Thoracic pain | 18 (8.61%) | 16 (15.7%) | 0.092^3^ |
| Palpitation | 24 (11.5%) | 21 (20.6%) | 0.049^3^ |
| Art. pain | 77 (36.8%) | 42 (41.2%) | 0.539^3^ |
| Myalgia | 100 (47.8%) | 49 (48.0%) | 1.000^3^ |
| Headache | 102 (48.8%) | 47 (46.1%) | 0.741^3^ |
| Head sensory comp. | 0 (0.00%) | 2 (1.96%) | 0.107^3^ |
| Sensory comp. | 41 (19.6%) | 16 (15.7%) | 0.493^3^ |
| Talk dis. | 14 (6.70%) | 6 (5.88%) | 0.977^3^ |
| Ear dis. | 31 (14.8%) | 14 (13.7%) | 0.929^3^ |
| Nausea | 17 (8.13%) | 11 (10.8%) | 0.578^3^ |
| Diarrhoea | 37 (17.7%) | 15 (14.7%) | 0.615^3^ |
| Anosmia/ageusia | 42 (20.1%) | 49 (48.0%) | <0.001^3^ |
| Fever | 46 (22.0%) | 29 (28.4%) | 0.271^3^ |
| Fatigue | 127 (60.8%) | 75 (73.5%) | 0.037^3^ |
| Memory comp. | 65 (31.1%) | 44 (43.1%) | 0.050^3^ |
| Attention comp. | 63 (30.1%) | 46 (45.1%) | 0.014^3^ |
| Concentration comp. | 84 (40.2%) | 54 (52.9%) | 0.045^3^ |
| Vertigo | 23 (11.0%) | 19 (18.6%) | 0.095^3^ |
| Sleep dis. | 95 (45.5%) | 53 (52.0%) | 0.338^3^ |
| Dermatological comp | 22 (10.5%) | 12 (11.8%) | 0.893^3^ |
| General synd. | 44 (21.1%) | 29 (28.4%) | 0.194^3^ |
| Neurological synd. | 40 (19.1%) | 33 (32.4%) | 0.015^3^ |
| Thoracic synd. | 1 (0.48%) | 4 (3.92%) | 0.041^3^ |
| Rheumatological syn. | 34 (16.3%) | 20 (19.6%) | 0.568^3^ |

### Table S6 Correlation matrix of symptoms.

|  | Cough | Dyspnoea | Thoracic pain | Palp. | Articular  pain | Myalgia | Headache | Head  sensory dis. | Sensitive  dis. | Speech dis. | Ear dis. | Nausea | Diarrhoea | Anosmia  /ageusia | Fever | Asthenia | Memory comp. | Attention comp | Concentration comp | Vertigo | Sleep dis. | Dermatological comp. |
| --- | --- | --- | --- | --- | --- | --- | --- | --- | --- | --- | --- | --- | --- | --- | --- | --- | --- | --- | --- | --- | --- | --- |
| Cough | 1.00 |  |  |  |  |  |  |  |  |  |  |  |  |  |  |  |  |  |  |  |  |  |
| Dyspnoea | 0.26 | 1.00 |  |  |  |  |  |  |  |  |  |  |  |  |  |  |  |  |  |  |  |  |
| Thoracic pain | 0.18 | 0.35 | 1.00 |  |  |  |  |  |  |  |  |  |  |  |  |  |  |  |  |  |  |  |
| Palpitation | 0.03 | 0.26 | 0.12 | 1.00 |  |  |  |  |  |  |  |  |  |  |  |  |  |  |  |  |  |  |
| Articular pain | 0.12 | 0.14 | 0.11 | 0.09 | 1.00 |  |  |  |  |  |  |  |  |  |  |  |  |  |  |  |  |  |
| Myalgia | 0.20 | 0.21 | 0.16 | 0.04 | 0.33 | 1.00 |  |  |  |  |  |  |  |  |  |  |  |  |  |  |  |  |
| Headache | 0.14 | 0.12 | 0.06 | 0.04 | 0.03 | 0.18 | 1.00 |  |  |  |  |  |  |  |  |  |  |  |  |  |  |  |
| Head sensory dis | -0.05 | 0.06 | 0.10 | -0.03 | 0.02 | 0.08 | 0.08 | 1.00 |  |  |  |  |  |  |  |  |  |  |  |  |  |  |
| Sensitive disorder | 0.02 | 0.04 | 0.10 | 0.18 | 0.19 | 0.19 | 0.09 | 0.07 | 1.00 |  |  |  |  |  |  |  |  |  |  |  |  |  |
| Speech disorder | 0.04 | 0.09 | 0.12 | 0.00 | 0.17 | 0.04 | 0.04 | 0.14 | 0.08 | 1.00 |  |  |  |  |  |  |  |  |  |  |  |  |
| Ear disorder | 0.01 | -0.01 | 0.06 | 0.06 | 0.11 | 0.04 | 0.01 | 0.20 | 0.09 | 0.15 | 1.00 |  |  |  |  |  |  |  |  |  |  |  |
| Nausea | 0.13 | 0.11 | 0.25 | 0.13 | 0.17 | 0.13 | 0.17 | 0.12 | 0.03 | 0.05 | 0.19 | 1.00 |  |  |  |  |  |  |  |  |  |  |
| Diarrhoea | 0.13 | 0.10 | 0.06 | 0.09 | 0.11 | 0.05 | 0.12 | -0.04 | 0.08 | -0.08 | -0.06 | 0.22 | 1.00 |  |  |  |  |  |  |  |  |  |
| Anosmia/ageusia | 0.01 | 0.08 | 0.02 | 0.04 | -0.07 | -0.05 | -0.12 | -0.05 | -0.01 | 0.00 | -0.08 | 0.02 | 0.03 | 1.00 |  |  |  |  |  |  |  |  |
| Fever | 0.39 | 0.24 | 0.14 | 0.02 | 0.05 | 0.24 | 0.30 | 0.05 | 0.06 | 0.07 | -0.04 | 0.11 | 0.19 | 0.08 | 1.00 |  |  |  |  |  |  |  |
| Asthenia | 0.21 | 0.27 | 0.11 | 0.03 | 0.12 | 0.23 | 0.23 | 0.06 | 0.07 | 0.03 | -0.02 | 0.16 | 0.13 | -0.12 | 0.38 | 1.00 |  |  |  |  |  |  |
| Memory complaint | 0.00 | 0.16 | 0.17 | 0.16 | 0.23 | 0.08 | 0.04 | 0.03 | 0.12 | 0.19 | 0.14 | 0.10 | 0.10 | 0.02 | 0.07 | 0.21 | 1.00 |  |  |  |  |  |
| Attention complaint | 0.09 | 0.26 | 0.22 | 0.16 | 0.23 | 0.17 | 0.05 | 0.03 | 0.09 | 0.22 | 0.02 | 0.12 | 0.09 | 0.02 | 0.18 | 0.26 | 0.52 | 1.00 |  |  |  |  |
| Concentration complaint | 0.07 | 0.22 | 0.21 | 0.15 | 0.23 | 0.14 | 0.10 | 0.01 | 0.08 | 0.16 | 0.09 | 0.17 | 0.19 | -0.01 | 0.15 | 0.26 | 0.47 | 0.74 | 1.00 |  |  |  |
| Vertigo | 0.11 | 0.28 | 0.22 | 0.10 | 0.17 | 0.20 | 0.17 | 0.09 | 0.18 | 0.05 | 0.16 | 0.27 | 0.02 | 0.06 | 0.11 | 0.21 | 0.20 | 0.28 | 0.23 | 1.00 |  |  |
| Sleep disorder | 0.03 | 0.03 | 0.04 | 0.10 | 0.20 | 0.14 | 0.17 | 0.00 | 0.21 | 0.01 | 0.01 | 0.04 | 0.13 | -0.06 | 0.06 | 0.11 | 0.15 | 0.12 | 0.24 | 0.15 | 1.00 |  |
| Dermatological complaint | 0.01 | 0.10 | 0.04 | 0.06 | 0.11 | 0.08 | 0.06 | 0.10 | 0.10 | 0.12 | 0.09 | 0.07 | 0.01 | -0.04 | 0.00 | 0.00 | 0.02 | 0.02 | 0.08 | 0.16 | 0.08 | 1.00 |

### Figure S1 Correlation analysis of persistent symptoms in PASC. The thickness of the link indicates the strength of the association. Symptoms usually associated to a system are of the same colour.

Figure S2 Association between PASC status and biomarker levels: Results of the multivariable analysis of the overall population comparing participants with PASC and those who recovered; subgroup analysis by the time from infection. P-values <0.05 are un bold. The most conservative significance threshold remained unmet.****

.

Figure S3 Associations between each persistent symptom and biomarker levels: Results of the multivariable analysis of the overall population adjusted for time from infection. Significant values are coloured depending on different significant level thresholds. The most conservative significance threshold remained unmet.

Figure S4 Associations between each persistent symptom and biomarker levels: Results of the multivariable analysis of the participants with the last infection having occurred less than one year before the study. Significant values are coloured depending on different significant level thresholds.

Figure S5 Associations between each persistent symptom and biomarker levels: Results of the multivariable analysis of the participants with the last infection having occurred more than one year before the study. Significant values are coloured depending on different significant level thresholds. The most conservative significance threshold remained unmet.

Figure S6 Association between medically-confirmed-PASC and biomarker levels: Results of the multivariable analysis of the overall population; subgroup analysis by the time from infection. None of the p-values reached the significance threshold for multiple testing. None of the p-values reached the significance threshold for multiple testing.

Figure S7 Association between each persistent symptom in the medically-confirmed-PASC subgroup and biomarker level: Results of the multivariable analysis of the overall population. Significant values are coloured depending on different significant level thresholds. None of the p-values reached the significance threshold for multiple testing.

Figure S8 Association between each persistent symptom in the subgroup of medically-confirmed-PASC and biomarker level: Results of the multivariable analysis of participants whose time between blood sample collection and SARS-CoV-2 infection was longer than one year. Significant values are coloured depending on different significant level thresholds. The most conservative significance threshold remained unmet.

Figure S9 Association between each persistent symptom in the subgroup of medically-confirmed-PASC and biomarker level: Results of the multivariable analysis of participants whose time between blood sample and SARS-CoV-2 infection was less than one year. Significant values are in bold.

### Figure S10 Proportion of resolution of symptoms between M0 and M6 among participants with PASC depending on time from infection: A) All PASC; B) PASC with infection lasting less than one year prior to M0; C) PASC with infection lasting more than one year prior to M0. Proportion of persistent symptoms at M6 are shown in percentage. comp: complaints; dis: disorder.

#####

### Figure S11 Variation in biomarkers among Recovered-PASC participants at M6 and persistent PASC participants at M6. Significant values are in bold. None of the p-values reached the significance threshold for multiple testing.

**Table S7 Association between biomarkers and symptoms resolution** a) association between biomarker levels measured at M6 and symptom resolution at M6; b) association between the variation of biomarker levels between M0 and M6 and symptom resolution at M6. Only results with p value <=0.05 are shown. The most conservative significance threshold remained unmet.

| **a) Biomarker levels at M6** | | | | **b) Variation of biomarkers between M0 and M6** | | | |
| --- | --- | --- | --- | --- | --- | --- | --- |
| **Infection lasting less than one year before M0** | | | | **Infection lasting less than one year before M0** | | | |
| **Symptom** | **Biomarker** | **Estimate [95% CI]** | **P-value** | **Symptom** | **Biomarker** | **Estimate [95% CI]** | **P-value** |
| Fatigue | PD-L1 | 1.16 [0.38, 1.95] | 0,01314 | Fatigue | TNFa | -1.41 [-2.27, -0.55] | 0,00909 |
|  |  |  |  |  | ICAM-1 | -1.14 [-1.96, -0.33] | 0,02044 |
|  |  |  |  |  | VCAM-1 | -1.45 [-2.60, -0.31] | 0,03205 |
| **Infection lasting more than one year before M0** | | | | **Infection lasting more than one year before M0** | | | |
| **Symptom** | **Biomarker** | **Estimate [95% CI]** | **P-value** | **Symptom** | **Biomarker** | **Estimate [95% CI]** | **P-value** |
| Anosmia/ageusia | ICAM-1 | -1.10 [-1.84, -0.35] | 0,00738 | Anosmia/ageusia | IL-18 | 0.92 [0.40, 1.45] | 0,00126 |
|  | FABP | -0.85 [-1.65, -0.05] | 0,04866 |  | PD-L1 | 0.59 [0.03, 1.15] | 0,0459 |
| Cough | VCAM-1 | -0.78 [-1.40, -0.17] | 0,01642 | Anosmia/ageusia | TNFa | 0.73 [0.18, 1.28] | 0,01277 |
| Dyspnea | IFNg | 1.76 [0.56, 2.96] | 0,01323 | Derm. comp. | CD163 | 1.36 [0.33, 2.38] | 0,03595 |
|  | TRAIL | 0.97 [0.14, 1.79] | 0,03455 |  | PD-L1 | 1.37 [0.65, 2.09] | 0,00735 |
| Thoracic pain | VCAM-1 | 1.80 [0.29, 3.32] | 0,04778 | Ear dis. | IL-18 | 0.96 [0.10, 1.82] | 0,04368 |
| Art. pain | CD163 | -0.55 [-1.02, -0.08] | 0,02547 |  | VCAM-1 | -1.03 [-1.87, -0.18] | 0,0287 |
|  | VCAM-1 | -0.77 [-1.27, -0.27] | 0,00386 | Memory comp. | IL-18 | 0.71 [0.27, 1.16] | 0,00284 |
| Ear dis. | IL-8 | -0.91 [-1.70, -0.13] | 0,03999 | Vertigo | TNFa | -1.06 [-1.78, -0.34] | 0,01052 |
| Diarrhoea | ICAM-1 | -1.36 [-2.47, -0.24] | 0,04099 |  |  |  |  |
|  | TRAIL | 1.13 [0.16, 2.10] | 0,04516 |  |  |  |  |
| Memory comp. | PD-L1 | -0.60 [-1.08, -0.12] | 0,01757 |  |  |  |  |
| Attention comp. | IFNg | 0.90 [0.08, 1.71] | 0,04701 |  |  |  |  |
| Overall | | | | Overall | | | |
| **Symptom** | **Biomarker** | **Estimate [95% CI]** | **P-value** | **Symptom** | **Biomarker** | **Estimate [95% CI]** | **P-value** |
| Anosmia/ageusia | ICAM-1 | -0.94 [-1.75, -0.13] | 0,03011 | Anosmia/ageusia | IL-18 | 0.63 [0.22, 1.05] | 0,00402 |
|  | FABP | -0.83 [-1.59, -0.08] | 0,04094 |  | PD-L1 | 0.56 [0.12, 1.00] | 0,01489 |
| Cough | CD163 | -0.55 [-1.07, -0.03] | 0,0431 |  | TNFa | 0.67 [0.24, 1.10] | 0,00325 |
|  | VCAM-1 | -0.77 [-1.31, -0.22] | 0,00833 |  | VCAM-1 | 0.64 [0.18, 1.10] | 0,00856 |
| Dyspnea | IFNg | 1.83 [0.58, 3.08] | 0,01323 | Art. pain | ICAM-1 | -0.45 [-0.87, -0.04] | 0,03602 |
|  | IL-18 | 0.99 [0.09, 1.90] | 0,04297 | Cough | IP-10 | 0.54 [0.05, 1.03] | 0,03536 |
|  | TRAIL | 1.12 [0.31, 1.94] | 0,01518 | Fatigue | VCAM-1 | -0.32 [-0.63, -0.01] | 0,04275 |
| Thoracic pain | VCAM-1 | 1.82 [0.36, 3.27] | 0,0367 | Memory comp. | IL-18 | 0.55 [0.16, 0.94] | 0,00697 |
| Art. pain | CD163 | -0.45 [-0.88, -0.03] | 0,04138 | Myalgia | IL-6 | -0.44 [-0.79, -0.08] | 0,01777 |
|  | VCAM-1 | -0.75 [-1.20, -0.29] | 0,00208 | Myalgia | VCAM-1 | -0.48 [-0.85, -0.11] | 0,01232 |
| Diarrhoea | TRAIL | 1.41 [0.38, 2.44] | 0,02157 | Nausea | IL-18 | 0.74 [0.08, 1.41] | 0,04115 |
| Fatigue | IFNg | 0.55 [0.02, 1.09] | 0,0493 | Nausea | IL-8 | 0.77 [0.06, 1.48] | 0,04714 |
| Memory comp. | PD-L1 | -0.67 [-1.12, -0.23] | 0,00454 | Sensory comp. | CD163 | -0.61 [-1.15, -0.07] | 0,03162 |
|  |  |  |  |  | IFNg | -0.82 [-1.34, -0.30] | 0,00362 |
|  |  |  |  |  | TRAIL | -0.59 [-1.13, -0.06] | 0,03644 |
|  |  |  |  | Vertigo | TRAIL | -0.85 [-1.58, -0.12] | 0,03026 |
